# Si_1–*x*_Ge_*x*_ anode synthesis on plastic films for flexible rechargeable batteries

**DOI:** 10.1038/s41598-022-18072-4

**Published:** 2022-08-12

**Authors:** H. Murata, K. Nozawa, T. Suzuki, Y. Kado, T. Suemasu, K. Toko

**Affiliations:** 1 Device Technology Research Institute, National Institute of Advanced Industrial Science and Technology (AIST), 1-1-1 Umezono, Tsukuba, Ibaraki 305-8568 Japan; 2 Institute of Applied Physics, University of Tsukuba, 1–1–1 Tennodai, Tsukuba, Ibaraki 305-8573 Japan; 3 Energy Process Research Institute, National Institute of Advanced Industrial Science and Technology (AIST), 16-1 Onogawa, Tsukuba, Ibaraki 305-8569 Japan

**Keywords:** Electrical and electronic engineering, Batteries

## Abstract

SiGe is a promising anode material for replacing graphite in next generation thin-film batteries owing to its high theoretical charge/discharge capacity. Metal-induced layer exchange (LE) is a unique technique used for the low-temperature synthesis of SiGe layers on arbitrary substrates. Here, we demonstrate the synthesis of Si_1−*x*_Ge_*x*_ (*x* = 0–1) layers on plastic films using Al-induced LE. The resulting SiGe layers exhibited high electrical conductivity (up to 1200 S cm^−1^), reflecting the self-organized doping effect of LE. Moreover, the Si_1−*x*_Ge_*x*_ layer synthesized by the same process was adopted as the anode for the lithium-ion battery. All Si_1−*x*_Ge_*x*_ anodes showed clear charge/discharge operation and high coulombic efficiency (≥ 97%) after 100 cycles. While the discharge capacities almost reflected the theoretical values at each *x* at 0.1 C, the capacity degradation with increasing current rate strongly depended on *x*. Si-rich samples exhibited high initial capacity and low capacity retention, while Ge-rich samples showed contrasting characteristics. In particular, the Si_1−*x*_Ge_*x*_ layers with *x* ≥ 0.8 showed excellent current rate performance owing to their high electrical conductivity and low volume expansion, maintaining a high capacity (> 500 mAh g^–1^) even at a high current rate (10 C). Thus, we revealed the relationship between SiGe composition and anode characteristics for the SiGe layers formed by LE at low temperatures. These results will pave the way for the next generation of flexible batteries based on SiGe anodes.

To sustain the further development of flexible electronics in the future, thin, lightweight, and flexible batteries are highly desirable^1^. Conventional Li-ion batteries (LIBs) use a liquid electrolyte, which inhibits flexibility and ease of handling. In recent years, research on all-solid-state batteries has become increasingly important for battery innovation. All-solid-state batteries have the advantages of high energy and power densities, good capacity retention for thousands of charge/discharge cycles, and high safety^2^, in addition to increasing the feasibility of flexible batteries.

Graphite has been used as an anode material in conventional LIBs with liquid electrolyte^3^. Although graphite is generally synthesized at high temperatures (> 2000 °C), we achieved low-temperature synthesis of graphite thin films (i.e., multilayer graphene (MLG)) on plastic films (polyimide: heat resistance of up to 400 °C) via metal-induced layer exchange (LE)^4^. In LE, an amorphous layer crystallizes through LE between the amorphous layer and metal catalyst layer^5,6^. LE will be suitable for microfabrication of devices because it can synthesize crystalline thin films with controlled thicknesses on arbitrary substrates at low temperatures^7,8^. Herein, we demonstrated the anode operation of MLG thin films synthesized by LE^4^; however, the capacity was limited to its low theoretical capacity of 372 mAh g^–1^^3^.

As new anode materials, IV group materials such as Si and Ge, which are well known in the field of semiconductors, are attracting increasing attention because of their high theoretical capacity^9–11^. While Si anodes have a considerably high theoretical capacity (e.g., 3580 mAh g^–1^ for Li_15_Si_4_), the high-rate capacity and cycle characteristics are limited by their low electrical and ionic conductivities^12–15^. Ge anodes, although inferior to Si in theoretical capacity (e.g., 1625 mAh g^–1^ for Li_4.4_Ge), exhibit good rate performance and cycle characteristics owing to their 100 times higher electrical conductivity (*σ*), 400 times higher Li^+^ diffusivity than Si, and lower volume expansion than Si^16–19^.

SiGe alloys are promising materials for anodes because they possess the advantageous properties of Si and Ge, while compensating for the disadvantages of the individual materials^20–30^. For example, it has been reported that the Si and Ge alloy alleviates the volumetric change during Li intercalation^21,27^. Although fabricating bulk SiGe requires a complicated process owing to its melting point difference, it is easy to prepare thin films because SiGe alloys are all-proportional solid solutions^31–33^. In general, SiGe requires high temperatures for impurity activation; therefore, high *σ* would not be obtained in low-temperature processes^34,35^. In our previous study, we overcame this problem using LE^34–38^. The SiGe thin film synthesized by Al-induced LE contains a metal equivalent to the solid solubility limit of SiGe; therefore, Al is self-organized and highly doped^34,36^. Therefore, the SiGe layers formed by LE exhibit high *σ*, even though the synthesis temperature is low (≤ 500 °C). Reflecting these features, the SiGe layers showed great potential for various applications, such as in thermoelectric devices^37,38^.

These characteristics of SiGe thin films formed by LE are also promising for the anodes of rechargeable batteries. In this study, we demonstrated the synthesis of SiGe thin films on plastic (polyimide) films using Al-induced LE over a wide range of Si_1–*x*_Ge_*x*_ compositions (*x* = 0, 0.15, 0.3, 0.6, 0.8, and 1) and clarified the basic LIB anode characteristics.

## Results and discussion

**Figure 1:**
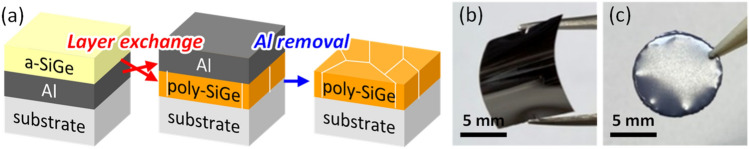
(**a**) Schematic of the Al-induced LE process. Photograph of the Si_0.7_Ge_0.3_ samples formed on the (**b**) plastic film and (**c**) Mo foil after Al removal.

Figure 1a shows a schematic of the Al-induced LE process. The LE mechanism is as follows. During annealing, Si and Ge atoms diffuse from the amorphous layer into the Al layer, mainly through the Al grain boundaries. When the Si and Ge concentration in Al is supersaturated, the SiGe nucleates in Al. After that, Si and Ge atoms dissolving in Al contact the nuclei, which induces lateral growth of SiGe. The lateral growth stresses Al and pushes it to the upper layer in a process called push-up phenomenon. Eventually, SiGe forms a bottom layer while Al forms an upper layer. A plastic (polyimide) film, covered with a SiO_2_ layer to avoid contamination to the SiGe layer^6^, was used as the substrate to evaluate the electrical properties, whereas Mo foil was used to evaluate the LIB anode characteristics. A stacked structure of a-Si_1−*x*_Ge_*x*_ (*x* = 0, 0.15, 0.3, 0.6, 0.8, and 1) on Al was formed on the substrates and subsequently annealed to induce LE. As shown in Fig. 1b,c, SiGe layers were formed on the flexible plastic film and Mo foil after Al removal. The plastic sample remains highly flexible, indicating that this anode synthesis process is useful for flexible rechargeable batteries.

**Figure 2:**
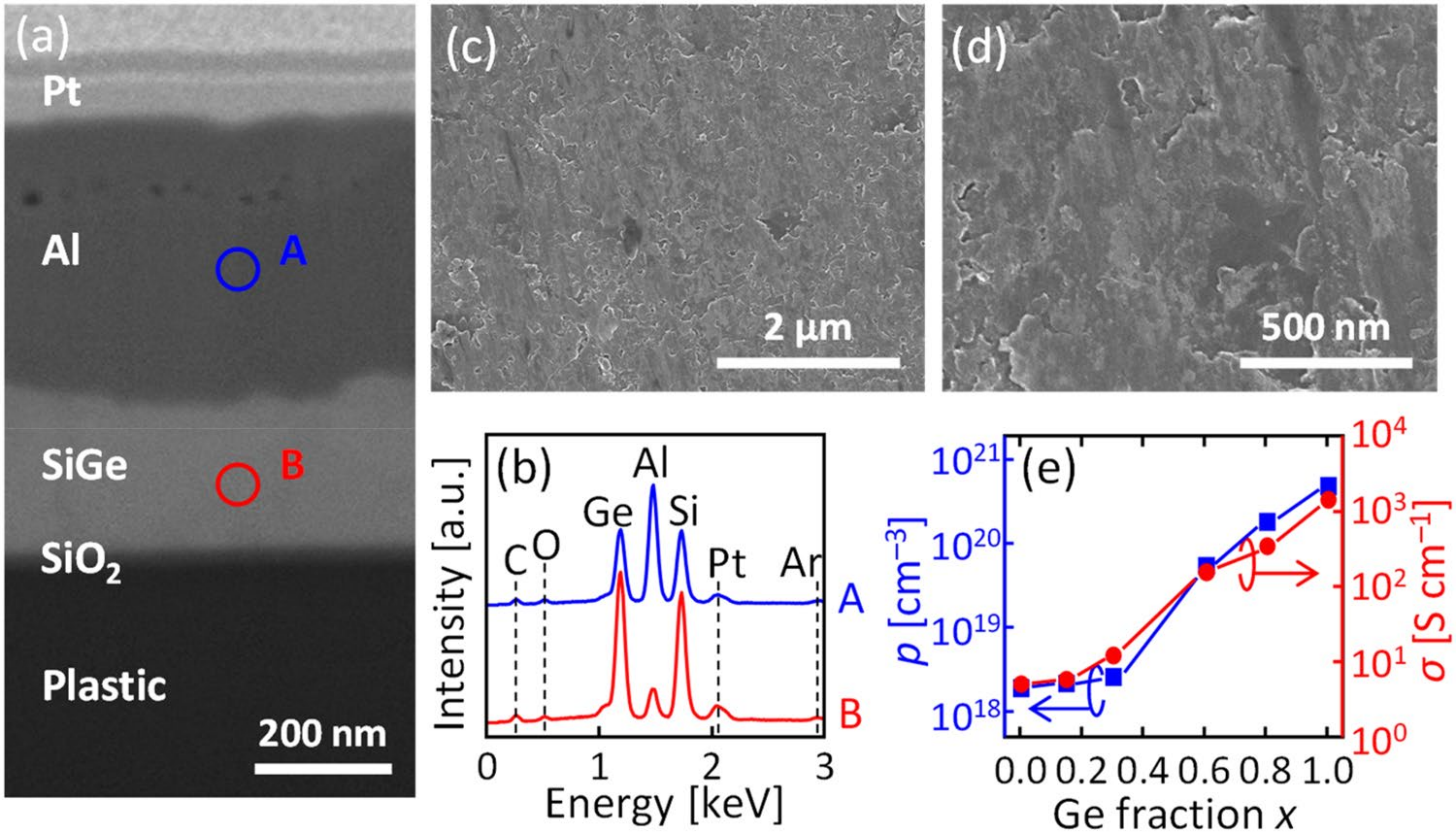
Characteristics of SiGe layers formed on a plastic film. (**a**) Cross-sectional FIB-SEM image of the Si_0.7_Ge_0.3_ sample tilted to 52°. (**b**) EDX spectra of the sample obtained at the circles A and B in (**a**). (**c**) Low and (**d**) high magnification SEM images of the Si_0.7_Ge_0.3_ sample after Al removal. (**e**) Hole concentration (*p*) and electrical conductivity (*σ*) of Si_1–*x*_Ge_*x*_ samples as a function of *x* (*x* = 0, 0.15, 0.3, 0.6, 0.8, and 1) after Al removal.

Figure 2a shows the scanning electron microscope (SEM) image of a sample using a plastic substrate, indicating a clear contrast of the stacked layer structure. The top Pt layer was prepared to protect the SiGe from the focused ion beam (FIB) process. Figure 2b shows the energy-dispersive X-ray (EDX) spectra at circles A and B shown in Fig. 2a, where the signals of the back and surrounding materials are also detected. The EDX spectra show that the positions of the Al and SiGe layers are exchanged. Figures 2c,d show that a continuous SiGe layer is formed on the plastic film after Al removal. The SiGe surface is rough, likely reflecting the initial Al shape, which was deposited by the sputtering method^6^. To evaluate the electrical properties of the obtained SiGe, Hall effect measurements were performed using the Van der Pauw method (Fig. 2e). All samples were found to be p-type because Al acts as an acceptor for SiGe. The hole concentration (*p*) increases with increasing Ge composition (*x*). This reflects the solid solubility limit of Al in each SiGe composition because the semiconductor film after LE contains metal equivalent to the solid solubility limit^6^. Reflecting the behavior of *p*, *σ* increases with increasing *x*. In semiconductor materials such as Si and Ge, impurity doping is necessary to increase *σ*, where a high *σ* is required for battery anodes. However, it is difficult to use non-thermal resistance substrates because high temperatures are required to activate the impurities. LE has the advantage of self-organization doping at low temperatures, which enables the use of non-thermal resistance substrates.

**Figure 3:**
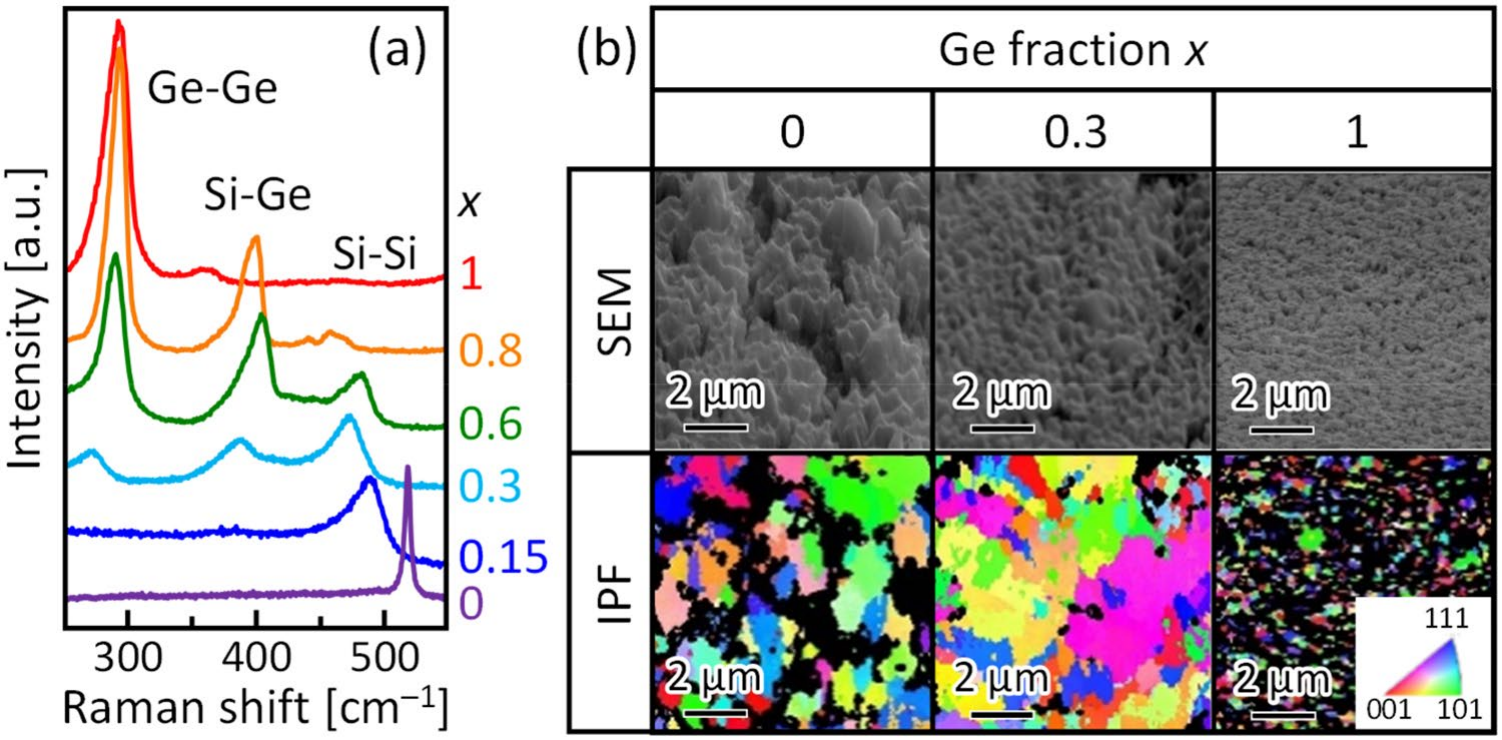
Characteristics of the SiGe layers formed on Mo foils after Al removal. (**a**) Raman spectra obtained from the Si_1−*x*_Ge_*x*_ (*x* = 0, 0.15, 0.3, 0.6, 0.8, and 1) samples. (**b**) SEM and IPF images of the 70° tilted Si_1−*x*_Ge_*x*_ (*x* = 0, 0.3, and 1) samples.

We evaluated the crystallinity of the SiGe layers on the Mo foil after Al removal. Figure 3a shows the peaks corresponding to the Si–Si, Si–Ge, and Ge–Ge vibrational modes, located at approximately 300, 400, and 500 cm^–1^, respectively, indicating that crystalline Si_1−*x*_Ge_*x*_ layers form on the substrates for all samples. The SiGe compositions, calculated from the Raman spectra, were the same as those of the as-prepared Si_1−*x*_Ge_*x*_ layers^39,40^. The surface morphology and crystallinity of the Si_1−*x*_Ge_*x*_ layers on the Mo foils were evaluated using SEM and electron backscattering diffraction (EBSD). The SEM images in Fig. 3b show that the sample surface is rough, which is a typical feature of LE that is also found on glass and plastics^6^. The inverse pole figure (IPF) images in Fig. 3b show that the SiGe layer is polycrystalline with random orientation. The grain size is smaller than that of the Si_1−*x*_Ge_*x*_ layers synthesized on a glass substrate. This is probably due to the use of Mo foil with a rough surface, considering that a rough substrate makes the grains smaller in LE^6^. Higher annealing temperature and higher Ge composition also produce smaller grains^6^. The different grain sizes in the current samples reflect these properties. Thus, Si_1−*x*_Ge_*x*_ layers were synthesized at low temperatures on Mo foil using LE.

**Figure 4:**
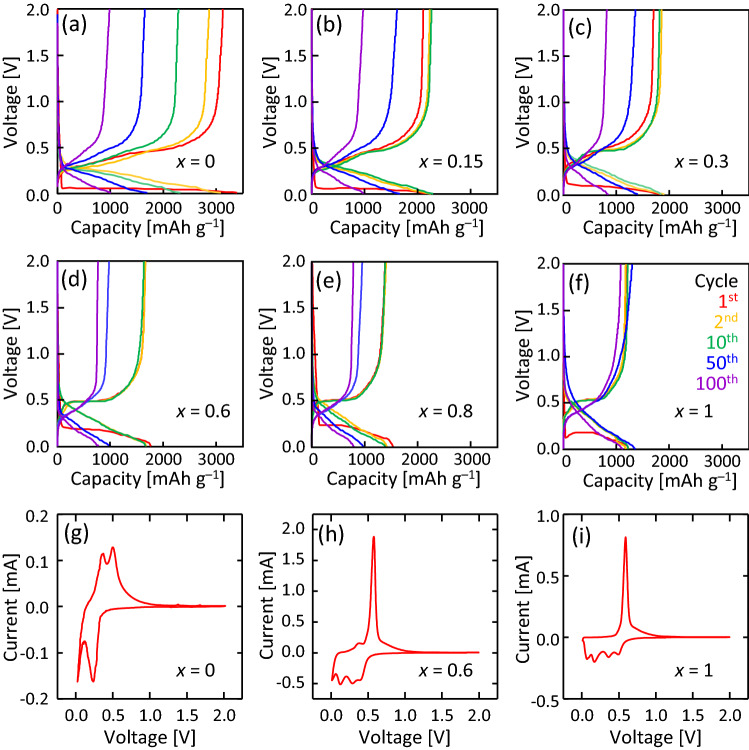
Electrochemical characteristics of the Si_1−*x*_Ge_*x*_ anode in the coin-type cell. Galvanostatic charge/discharge cycles of the sample at a current density of 1 C for *x* = (**a**) 0, (**b**) 0.15, (**c**) 0.3, (**d**) 0.6, (**e**) 0.8, and (**f**) 1. Cyclic voltammograms (CV) showing the fifth cycle at a scan rate of 100 μV s^–1^ for *x* = (g) 0, (h) 0.6, and (i) 1.

Figures 4a–f show that the coin-type cells using the Si_1–*x*_Ge_*x*_ layers exhibit a clear charge/discharge operation over 100 cycles for all *x*, where the capacity significantly depends on *x*. Figures 4g–i show that the cyclic voltammograms (CV) of the Si, Si_0.4_Ge_0.6_, and Ge samples exhibit various peaks due to the reaction with Li. The CV for Si (Fig. 4g) exhibited clear cathodic peaks (0.02 and 0.24 V) and anodic peaks (0.07, 0.17, 0.36, and 0.49 V) corresponding to lithium alloying/dealloying of Si. The analogous result was observed for Ge (Fig. 4i). According to literatures on lithiation mechanism of pure Si and Ge by in situ analyses such as Raman spectroscopy, XRD measurement, and TEM observation, several kinds of lithium compounds (e.g., LiSi, Li_7_Si_3_, Li_15_Si_4_, Li_9_Ge_4_, and Li_15_Ge_4_) were involved through charge/discharge processes^17,41–46^. The CV of Si_0.4_Ge_0.6_ (Fig. 4h) appeared an overlapped shape of Fig. 4g,i, indicating that lithiation mechanism of Si_0.4_Ge_0.6_ can be explained by the lithium alloying both of Si and Ge.

**Figure 5:**
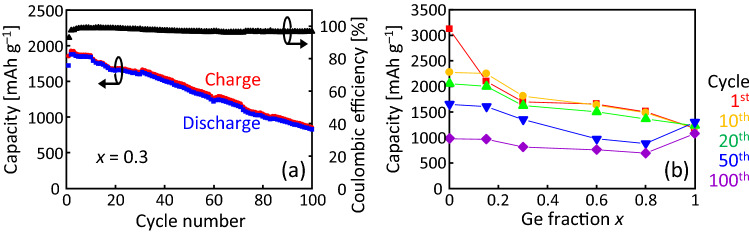
Electrochemical characteristics of the Si_1−*x*_Ge_*x*_ anode at a current density of 1 C. (**a**) Cycle number dependence of the discharge capacities and coulombic efficiency of the Si_1−*x*_Ge_*x*_ anode for *x* = 0.3. (**b**) Discharge capacity at 1, 10, 20, 50, and 100 cycles as a function of *x*.

Figure 5a shows a typical example of the cycle characteristics and coulombic efficiency calculated from the charge/discharge characteristics. The charge/discharge capacity gradually decreased as the cycle was repeated. This behavior is explained by the pulverization of the SiGe layer due to the expansion and contraction caused by Li charge/discharge^20,22^. The discharge capacity is 810 mAh g^–1^ after 100 cycles, which is 48% of the initial discharge capacity (1700 mAh g^–1^). The coulombic efficiency is relatively low in the first cycle due to irreversible capacity formation but maintains a high value of 97% in the following cycles. Figure 5b shows that the discharge capacity and its cycle characteristics, obtained from the charge/discharge characteristics (Fig. 4a–f), significantly depend on *x*. The Si-rich samples showed high initial capacity and low capacity retention, while the Ge-rich samples showed low initial capacity and high capacity retention. The *x* dependence of the anode properties is reasonable, considering the results of CV (Fig. 4g–i) and that Si has a higher Li storage capacity and volume expansion during the Li insertion than Ge^19,25,47^.

**Figure 6:**
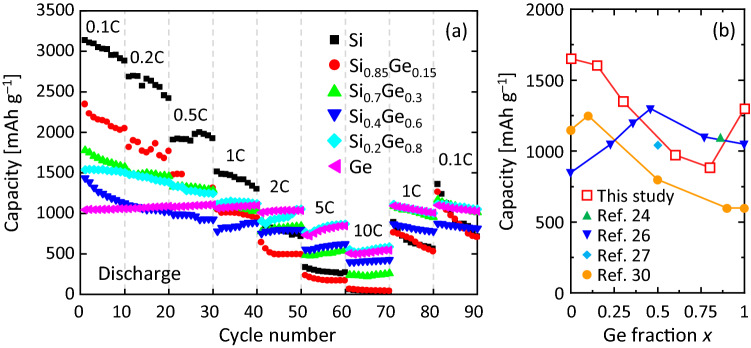
(**a**) Current-rate testing ranged from 0.1 to 10 C, every 10 cycles of the Si_1−*x*_Ge_*x*_ (*x* = 0, 0.15, 0.3, 0.6, 0.8, and 1) anode in the coin-type cell. (**b**) Comparison of the Si_1−*x*_Ge_*x*_ anode capacity at the 50th cycle. The current density was set to 1 C, 1 C, 0.8 A g^–1^, 1 A g^–1^ and 2 A g^–1^ for this study, Ref. 24, Ref. 26, Ref. 27, and Ref. 30, respectively.

Figure 6a shows the current rate characteristics of the discharge capacity. Similar to the general anode, the capacity decreased with increasing current rate, which became more remarkable for smaller *x* values. The discharge capacities generally reflected the theoretical capacities for each *x* at 0.1 C, while the magnitude relationship with respect to *x* was reversed at higher current rates (≥ 2 C). In particular, the discharge capacity at 10 C was below 50 mAh g^–1^ for *x* = 0 and 0.15, while it remained above 500 mAh g^–1^ for *x* = 0.8 and 1. In addition, the measurement results at 1 C and 0.1 C after 10 C showed good capacity recovery with increasing *x*. Therefore, in general, the anode material with a higher volume expansion rate is more remarkable in pulverization during high current rate charge/discharge^48,49^; this behavior mainly reflects the decrease in volume expansion rate with increasing *x*. The high *σ* at high *x* (Fig. 2c) and the film structure including voids (Fig. 3b) may also contribute to the excellent current rate performance of the Ge-rich SiGe layer. Figure 6b compares the capacity at the 50th cycle of Si_1−*x*_Ge_*x*_ LIB anodes formed using various techniques^24,26,27,30^. The SiGe formed in this study exhibited anode properties comparable to those of nanostructured SiGe in the entire SiGe composition range. Therefore, LE has an advantage over the conventional method in that it allows large-area SiGe formation in a simple low-temperature process. Optimizing the cathode and electrolyte will lead to the demonstration of a full cell with excellent properties.

## Conclusions

We demonstrated the low-temperature synthesis of SiGe layers using Al-induced LE, and investigated their anode characteristics to realize flexible rechargeable batteries. We demonstrated the synthesis of Si_1−*x*_Ge_*x*_ (*x* = 0, 0.15, 0.3, 0.6, 0.8, and 1) thin films on plastic (polyimide) films (≤ 400 °C). The LE-synthesized SiGe layer showed high *σ* due to self-organized doping, clear charge/discharge operation, and high coulombic efficiency (≥ 97%) after 100 cycles. The discharge capacities approached the theoretical capacities for each *x* at 0.1 C. The Si-rich samples showed higher initial capacity and lower capacity retention, while Ge-rich samples exhibited the opposite behavior. The capacity degradation with increasing current rate and capacity retention is highly dependent on *x*. SiGe with *x* ≥ 0.8 showed excellent current rate performance due to its *σ* and low volume expansion, maintaining a capacity of over 500 mAh g^–1^ even at 10 C. Therefore, we clarified the relationship between the SiGe composition and anode characteristics of the SiGe synthesized by Al-induced LE at low temperatures. This achievement will enhance the feasibility of flexible rechargeable batteries based on group IV material anodes.

## Methods

### Poly-SiGe preparation

The 250-nm-thick Al and 500-nm-thick Si_1–*x*_Ge_*x*_ (*x* = 0, 0.15, 0.3, 0.6, 0.8, and 1) layers were sequentially prepared on a polyimide film covered by a 30-nm-thick SiO_2_ layer. All depositions were performed by radio-frequency (RF) magnetron sputtering (base pressure: 3.0 × 10^–4^ Pa) with Ar plasma. The RF power was set at 100 W. The samples were annealed at 400 °C (20 h) for *x* = 0 and 375 °C (20 h) for *x* = 0.15, 0.3, 0.6, 0.8, and 1 in an N_2_ ambient chamber. The Al layer was then etched away using an hydrofluoric acid (HF) solution (HF 1.5%) for 2 min.

### Cell fabrication

A Mo foil (thickness: 50 μm) was used instead of a SiO_2_-covered polyimide film for electrochemical measurements. The SiGe preparation process was the same as that used for the polyimide substrate. The sample was punched through a 10-mm-diameter disk and then used as electrodes after being dried for 1 h under vacuum. The mass of SiGe was determined to be 85 μg for *x* = 0, 101 μg for *x* = 0.15, 143 μg for *x* = 0.3, 168 μg for *x* = 0.6, 185 μg for *x* = 0.8, and 214 μg for *x* = 1. Coin-type cells were fabricated from the SiGe electrode, pure Li metal foil, and separator (Celgard 2400), and subsequently immersed in an electrolyte. The electrolyte used was 1 mol L^−1^ of lithium hexafluorophosphate (LiPF_6_) in 1:1 ethylene carbonate (EC)/diethyl carbonate (DEC).

### Sample characterization

Hall effect measurements were performed using the Van der Pauw method using the Bio-Rad HL5500PC system. The sample structure was evaluated via SEM (Hitachi High-Technologies SU-8020), equipped with EDX, EBSD, and FIB-SEM (FEI Helios NanoLab 600i). The crystal quality and structure of SiGe were evaluated using Raman spectroscopy (JASCO NRS-5100, wavelength: 532 nm, spot diameter: 20 μm). The electrochemical characteristics of the samples were investigated using a multichannel galvanostat-potentiostat (Bio-Logic VMP).
